# Supplementary material for: Migration through a small pore disrupts inactive chromatin organization in neutrophil-like cells
Source: BMC Biol. 2018 Nov 26;16:142. doi: 10.1186/s12915-018-0608-2 (PMC6257957; doi:10.1186/s12915-018-0608-2)
Supplement: Supplementary file 1 — Figures S1–S6. 1. HL60 cells differentiated into neutrophil-like cells and migrated through two pore sizes. 2. SEM of porous membranes. 3. RNA-seq summary plots, and comparison of migration with differentiation. 4. Topological domain changes after migration through two pore sizes. 5. Compartment status of increased frequency contacts between migration with and without constriction. 6. Genome-wide distribution of disrupted contacts, compartments, and TADs. (PDF 3721 kb) [file 12915_2018_608_MOESM1_ESM.pdf]

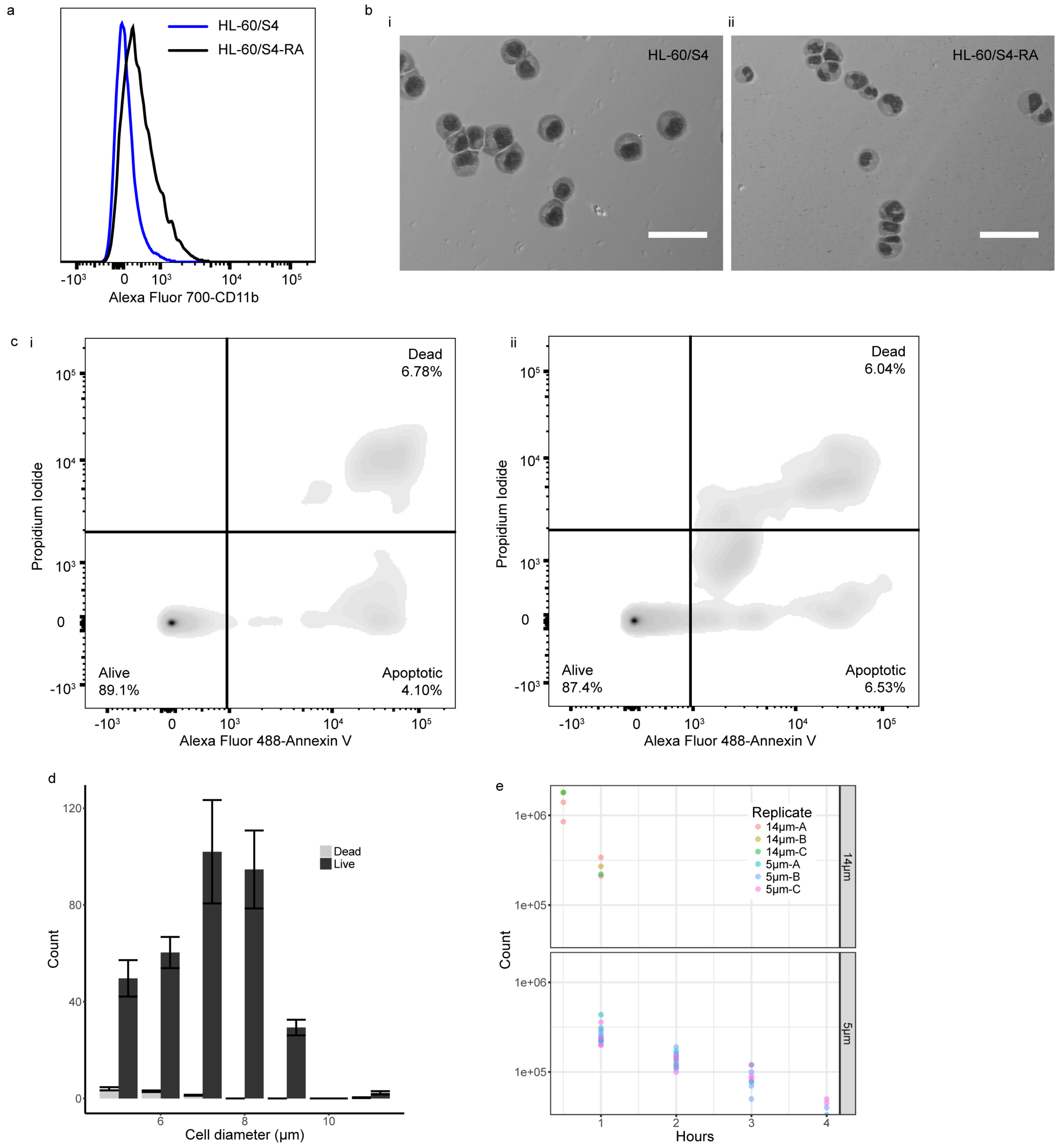

Supplementary Figure 1. *HL60 cells differentiated into neutrophil-like cells and migrated through two pore sizes.* a) Flow cytometry analysis of expression of the CD11b marker on HL60/S4 and HL60/S4-RA cells. HL-60/S4 cells treated with RA had higher levels of CD11b, indicating successful differentiation. Representative histogram shown. b) HL60 cells were cytospun onto microscope slides and Wright-Giemsa stained to compare nuclear morphology. Undifferentiated cells treated with vehicle (ethanol) for four days had spherical nuclei (i), while cells differentiated with retinoic acid for 4 days had lobed or partially lobed nuclei (ii). Representative images shown. c) Flow cytometry analysis of Alexa Fluor 488-annexin V and propidium iodide (PI) staining of live, dead and apoptotic cells following differentiation with retinoic acid (RA) (i) undifferentiated HL-60/S4 and (ii) differentiated HL-60/S4-RA cells. Over 85% of both HL-60/S4 and HL-60/S4-RA were viable. d) The JuliBr cell counting system (automated hemocytometer) was used to count and measure the diameter of cells in suspension. Cells were stained with trypan blue to

distinguish live and dead cells. Histogram shows mean count of cell diameters across 3 independent differentiation assays. Viable cells had an average diameter of 6.99 µm (SD=0.327). e) Migration rate through 5 µm and 14 µm diameter pores. Cells were collected and processed every 30 minutes during the migration assay, and formaldehyde fixed cells were counted to assess migration rate. All time points were pooled per experiment, up to two experiments were pooled for Hi-C replicates of 14 µm pore migration, and up to four experiments were pooled for Hi-C replicates of 5 µm pore migration. Migration rates were consistent within and between replicates at each time point. Cells migrated more slowly through 5 µm pores when compared to migration though 14 µm pores, as they are required to remodel to fit through the smaller pore size.

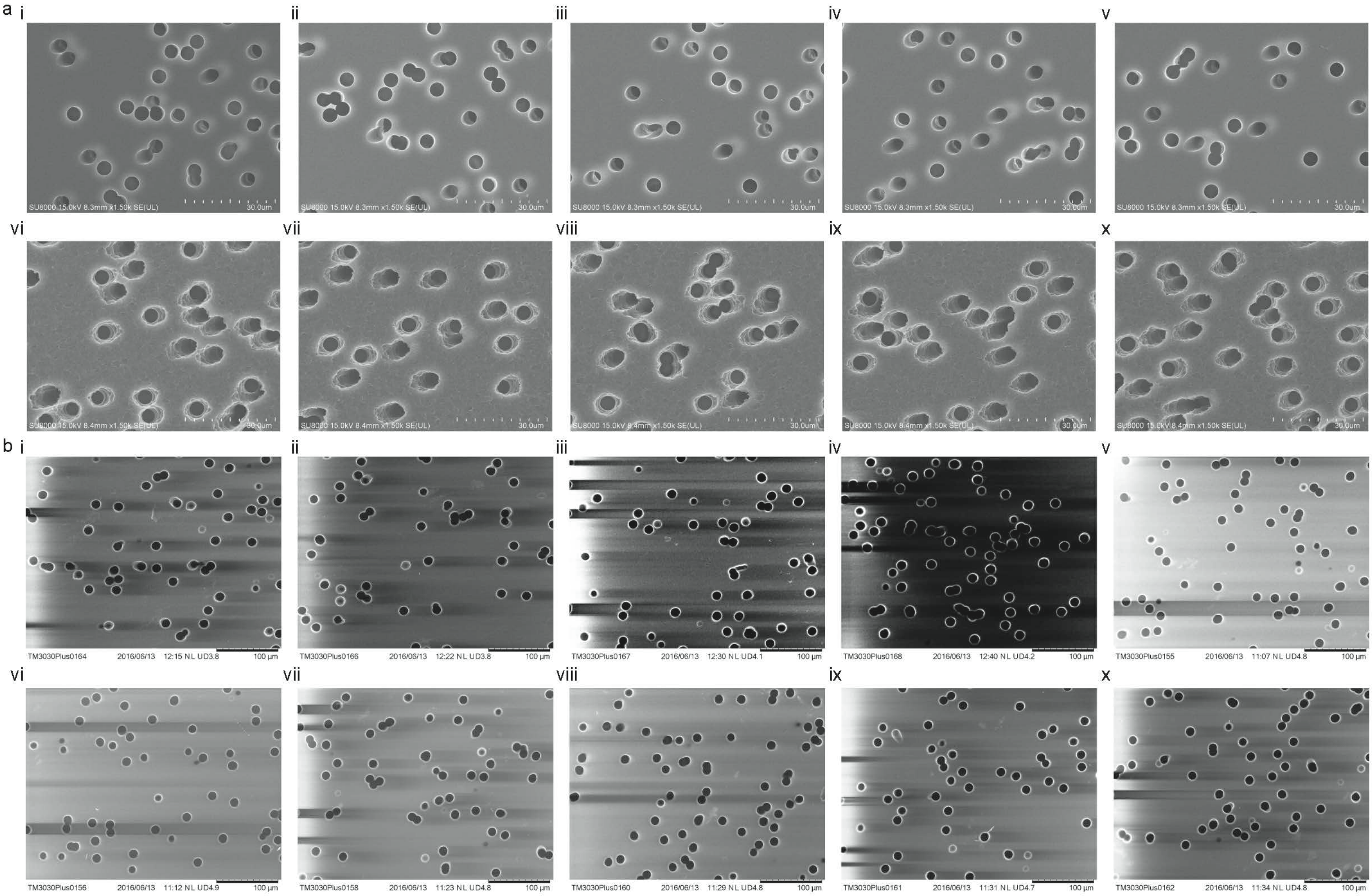

Supplementary Figure 2. *SEM of porous membranes.* a) Scanning electron microscopy of the smooth (i-v) and rough (vi-x) sides of polycarbonate membranes with 5µm diameter pores. a) Scanning electron microscopy of the rough (i-iv) and smooth (v-x) sides of polycarbonate membranes with 14µm diameter pores. Although both membranes contained pores that were joined to make a larger single pore, the majority were joined at the edges and thus did not increase the minimum diameter of the pore.

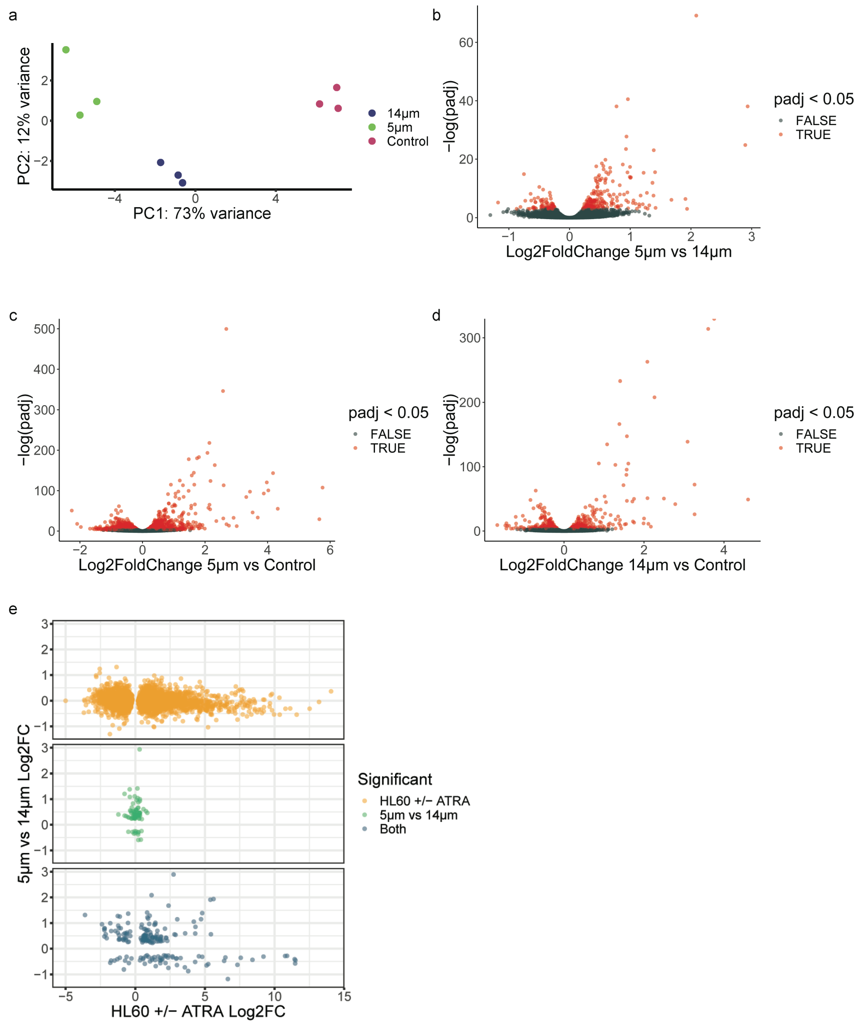

Supplementary Figure 3. *RNA-seq summary plots, and comparison of migration with differentiation.* a) VST normalised RNA-seq results cluster by condition. b) Volcano plot of FDR adjusted p-value and Log2 fold change in gene expression levels between migration with and without constriction. c) Volcano plot of FDR adjusted p-value and Log 2 Fold Change in gene expression levels between migration with constriction and unmigrated control cells. d) Volcano plot of FDR adjusted p-value and Log 2 Fold Change in gene expression levels between migration without constriction and unmigrated control cells. e) Gene expression changes between migration with and without constriction did not correlate with gene expression changes after differentiation with all-trans retinoic acid for 4 days [1]. The top panel shows the Log2 fold changes of genes that were significantly differentially expressed after differentiation, but not between migration with and without constriction (R^2=0.017, p=2x10^{-16}, y=-0.016x+0.001). The middle panel shows the Log2 fold changes of genes that were significantly differentially expressed between migration with and without constriction, but not after differentiation (R^2=0.068, p=6.6x10^{-6}, y=-0.069x+0.40). The bottom panel shows the Log2 fold changes of genes that were significantly differentially expressed in both experiments (R^2=0.073, p=6.7x10^{-5}, y=-0.07x+0.38).

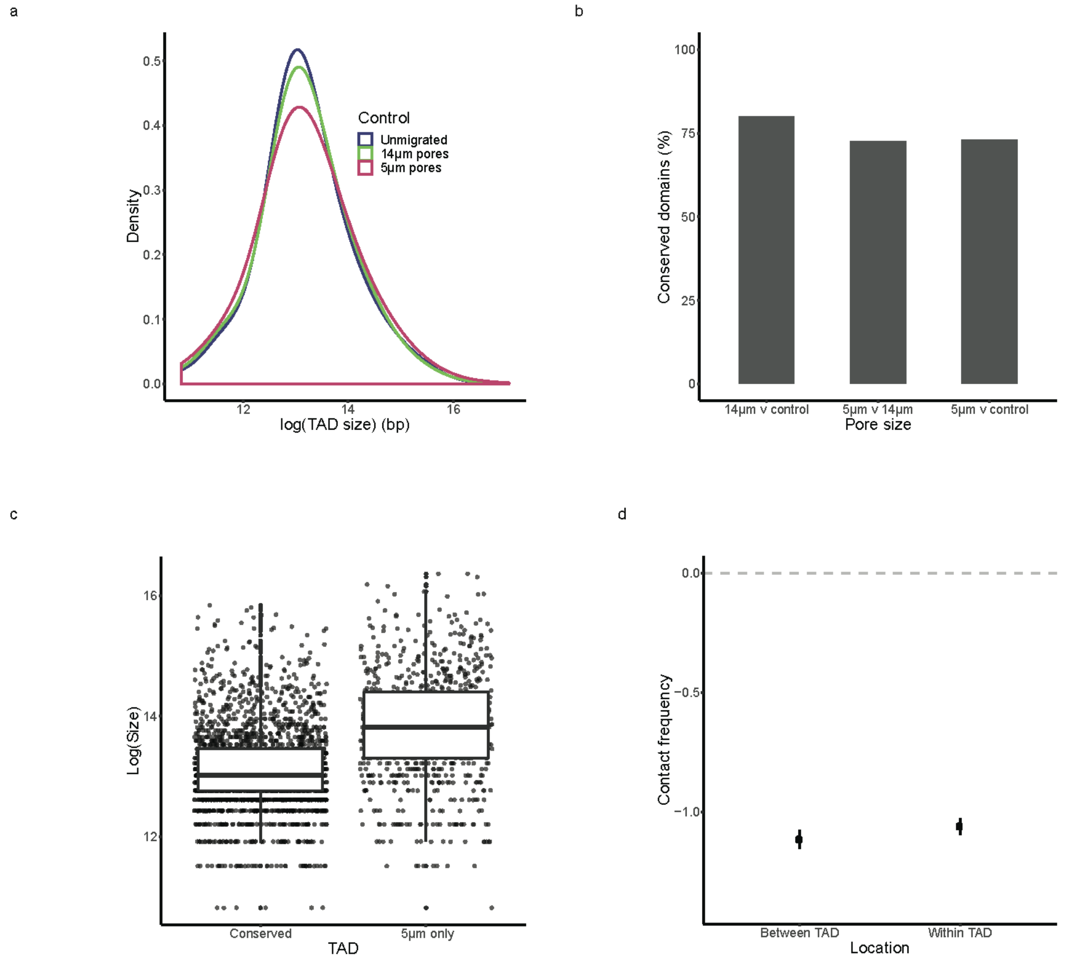

Supplementary Figure 4. *Topological domain changes after migration through two pore sizes.* a) TAD size after log transformation was not significantly different between control, 5µm migrated, and 14µm migrated cells (p>0.5, logistic regression). b) Domains were considered to have a conserved location if the condition location overlapped 80% or more with the control location. Domain location was most conserved between unmigrated and migrated without constriction. Domains were less conserved after migration with constriction. c) Domains that were conserved between migration with and without constriction were smaller than domains found only in migration with constriction ($p=2x10^{-16}$, $R^2=0.15$, logistic regression of log transformed TAD size), suggesting a loss of boundaries. d) Disruption of contacts occurred both within and between topologically associated domains (TADs). Contact frequency between upstream and downstream windows per bin (binsignal) as calculated by TopDom showed a statistically significant decrease after migration through 5µm pores, compared to migration through 14µm pores (Student's t test, all p values < 2.2e-16, difference in mean with 95% confidence displayed).

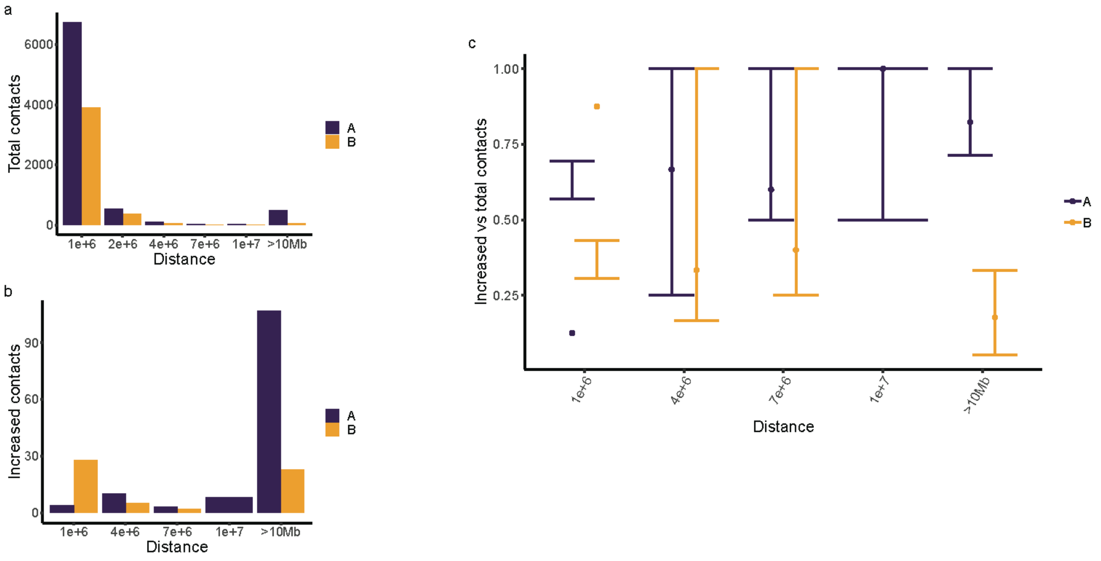

Supplementary Figure 5. *Compartment status of increased frequency contacts between migration with and without constriction.* Bin sizes are uneven. a) Number of significant contacts (FDR<0.05) in compartment A and B in cells migrated with constriction. b) Number of significantly increased contacts (FDR<0.1) in compartment A and B between migration with and without constriction. iii) Bootstrapping of significant contacts found a strong enrichment of increased short range (<1Mb) contacts in compartment B. Error bars represent 99% CI of the expected proportion of contacts in compartment A or B based on 1000 bootstraps.

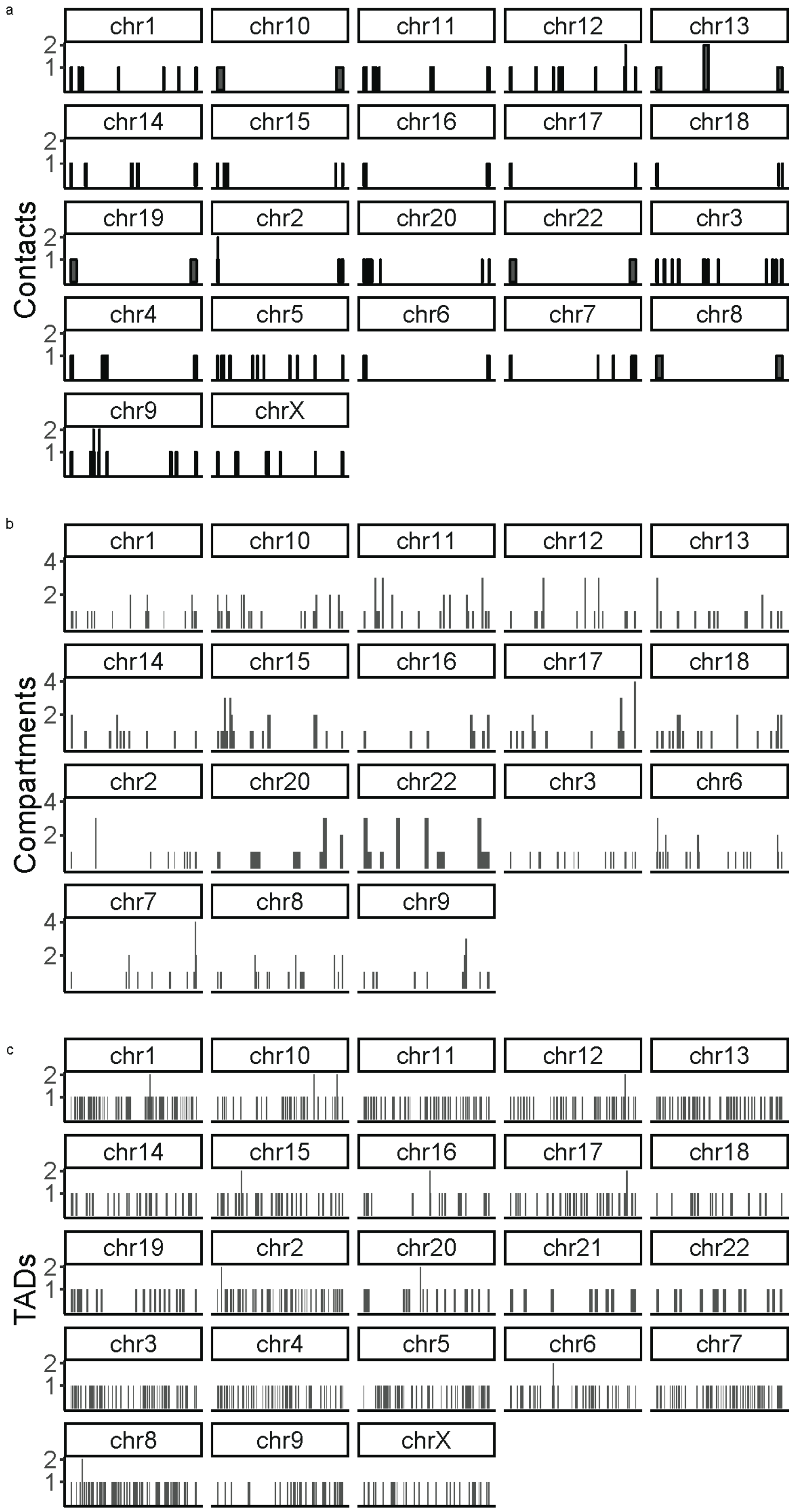

Supplementary Figure 6. *Genome wide distribution of disrupted contacts, compartments, and TADs.* a) There were no more than two differential contacts (FDR<0.1) between migration with and without constriction per megabase across the genome. b) There were no more than four

compartment disruptions (R<0.6, opposite PC1 value) between migration with and without constriction per megabase across the genome. b) There were no more than two non-conserved TADs (location <80% overlapping) between migration with and without constriction per megabase across the genome.
