# Supplementary material for: Migration through a small pore disrupts inactive chromatin organization in neutrophil-like cells
Source: BMC Biol. 2018 Nov 26;16:142. doi: 10.1186/s12915-018-0608-2 (PMC6257957; doi:10.1186/s12915-018-0608-2)
Supplement: Supplementary file 3 — Table S2-S5, S7-S9. 2. Gene ontology enrichment (Biological process) of Fig. 2b. 3. Gene ontology enrichment (Biological process) of Fig. 2c. 4. Gene ontology enrichment (Biological process) of Fig. 2d. 5. Gene ontology enrichment (Molecular function) of Fig. 2d. 7. RNA-seq QC. 8. HiCUP QC. 9. Hi-C replicate correlations. (DOCX 40 kb) [file 12915_2018_608_MOESM3_ESM.docx]

**Table S2.** Gene ontology enrichment (Biological process) of Figure 2b (set 1)

| GO.ID | Term | Annotated | Significant | Expected | weight01Fisher | FDR |
| --- | --- | --- | --- | --- | --- | --- |
| GO:0034097 | response to cytokine | 626 | 49 | 21.18 | 2.20E-08 | 4.06E-05 |
| GO:0000122 | negative regulation of transcription fro... | 513 | 41 | 17.36 | 8.70E-07 | 0.00080258 |
| GO:0045944 | positive regulation of transcription fro... | 658 | 52 | 22.27 | 2.00E-06 | 0.00123 |
| GO:0051591 | response to cAMP | 47 | 8 | 1.59 | 6.60E-05 | 0.02515909 |
| GO:0071347 | cellular response to interleukin-1 | 45 | 8 | 1.52 | 7.60E-05 | 0.02515909 |
| GO:0042149 | cellular response to glucose starvation | 33 | 7 | 1.12 | 9.50E-05 | 0.02515909 |
| GO:0042127 | regulation of cell proliferation | 832 | 59 | 28.16 | 9.60E-05 | 0.02515909 |
| GO:0071356 | cellular response to tumor necrosis fact... | 187 | 18 | 6.33 | 0.00012 | 0.02515909 |
| GO:0032496 | response to lipopolysaccharide | 182 | 28 | 6.16 | 0.00014 | 0.02515909 |
| GO:2000347 | positive regulation of hepatocyte prolif... | 4 | 3 | 0.14 | 0.00015 | 0.02515909 |
| GO:0090009 | primitive streak formation | 4 | 3 | 0.14 | 0.00015 | 0.02515909 |
| GO:0031663 | lipopolysaccharide-mediated signaling pa... | 42 | 10 | 1.42 | 0.00018 | 0.027675 |
| GO:0036499 | PERK-mediated unfolded protein response | 18 | 6 | 0.61 | 0.00023 | 0.03264231 |
| GO:0002548 | monocyte chemotaxis | 23 | 6 | 0.78 | 0.00035 | 0.04428 |
| GO:2000144 | positive regulation of DNA-templated tra... | 18 | 4 | 0.61 | 0.00036 | 0.04428 |
| GO:0032870 | cellular response to hormone stimulus | 391 | 25 | 13.23 | 5.00E-04 | 0.05535 |
| GO:0045766 | positive regulation of angiogenesis | 67 | 10 | 2.27 | 0.00053 | 0.05535 |
| GO:0032088 | negative regulation of NF-kappaB transcr... | 56 | 8 | 1.9 | 0.00054 | 0.05535 |
| GO:0032754 | positive regulation of interleukin-5 pro... | 6 | 3 | 0.2 | 0.00071 | 0.06413571 |
| GO:1902895 | positive regulation of pri-miRNA transcr... | 13 | 4 | 0.44 | 0.00072 | 0.06413571 |
| GO:0071277 | cellular response to calcium ion | 33 | 6 | 1.12 | 0.00073 | 0.06413571 |
| GO:0006954 | inflammatory response | 340 | 27 | 11.51 | 0.00083 | 0.06738261 |
| GO:0009615 | response to virus | 238 | 12 | 8.05 | 0.00084 | 0.06738261 |
| GO:0043434 | response to peptide hormone | 257 | 20 | 8.7 | 0.00097 | 0.06747429 |
| GO:0045682 | regulation of epidermis development | 33 | 6 | 1.12 | 0.00112 | 0.06747429 |
| GO:0043331 | response to dsRNA | 66 | 5 | 2.23 | 0.00114 | 0.06747429 |
| GO:0014839 | myoblast migration involved in skeletal ... | 2 | 2 | 0.07 | 0.00114 | 0.06747429 |
| GO:0002774 | Fc receptor mediated inhibitory signalin... | 2 | 2 | 0.07 | 0.00114 | 0.06747429 |
| GO:0061029 | eyelid development in camera-type eye | 7 | 3 | 0.24 | 0.00121 | 0.06747429 |
| GO:0071499 | cellular response to laminar fluid shear... | 7 | 3 | 0.24 | 0.00121 | 0.06747429 |
| GO:0051412 | response to corticosterone | 7 | 3 | 0.24 | 0.00121 | 0.06747429 |
| GO:0048715 | negative regulation of oligodendrocyte d... | 7 | 3 | 0.24 | 0.00121 | 0.06747429 |
| GO:0007623 | circadian rhythm | 112 | 13 | 3.79 | 0.00122 | 0.06747429 |
| GO:0071222 | cellular response to lipopolysaccharide | 107 | 18 | 3.62 | 0.00125 | 0.06747429 |
| GO:0043124 | negative regulation of I-kappaB kinase/N... | 42 | 7 | 1.42 | 0.00128 | 0.06747429 |
| GO:0006335 | DNA replication-dependent nucleosome ass... | 26 | 5 | 0.88 | 0.00158 | 0.080975 |
| GO:0031665 | negative regulation of lipopolysaccharid... | 9 | 4 | 0.3 | 0.00188 | 0.08893846 |
| GO:0070098 | chemokine-mediated signaling pathway | 27 | 5 | 0.91 | 0.00188 | 0.08893846 |
| GO:0010818 | T cell chemotaxis | 14 | 4 | 0.47 | 0.00188 | 0.08893846 |
| GO:0032922 | circadian regulation of gene expression | 40 | 6 | 1.35 | 0.00208 | 0.09594 |
| GO:0044344 | cellular response to fibroblast growth f... | 74 | 6 | 2.5 | 0.00216 | 0.0972 |

Gene ontology term enrichment (Biological Process) of all genes significantly differentially expressed (FDR<0.05) in both 5µm pores compared to control, and 14µm pores compared to control.

**Table S3.** Gene ontology enrichment (Biological process) of Figure 2C (set 2)

| GO.ID | Term | Annotated | Significant | Expected | weight01Fisher | FDR |
| --- | --- | --- | --- | --- | --- | --- |
| GO:0006954 | inflammatory response | 340 | 96 | 53.76 | 1.50E-06 | 0.0042735 |
| GO:0000122 | negative regulation of transcription fro... | 513 | 120 | 81.12 | 1.50E-05 | 0.011396 |
| GO:0006955 | immune response | 1192 | 267 | 188.49 | 1.50E-05 | 0.011396 |
| GO:0070098 | chemokine-mediated signaling pathway | 27 | 15 | 4.27 | 1.60E-05 | 0.011396 |
| GO:0048661 | positive regulation of smooth muscle cel... | 38 | 18 | 6.01 | 6.70E-05 | 0.0381766 |
| GO:0045944 | positive regulation of transcription fro... | 658 | 149 | 104.05 | 8.20E-05 | 0.03893633 |
| GO:0002467 | germinal center formation | 13 | 8 | 2.06 | 9.80E-05 | 0.03917375 |
| GO:0032496 | response to lipopolysaccharide | 182 | 60 | 28.78 | 0.00011 | 0.03917375 |
| GO:0071347 | cellular response to interleukin-1 | 45 | 17 | 7.12 | 0.00016 | 0.05064889 |
| GO:0031663 | lipopolysaccharide-mediated signaling pa... | 42 | 17 | 6.64 | 0.00025 | 0.071225 |
| GO:0035589 | G-protein coupled purinergic nucleotide ... | 8 | 6 | 1.27 | 0.00032 | 0.071225 |
| GO:0071356 | cellular response to tumor necrosis fact... | 187 | 49 | 29.57 | 0.00034 | 0.071225 |
| GO:0007166 | cell surface receptor signaling pathway | 1480 | 306 | 234.03 | 0.00034 | 0.071225 |
| GO:0007565 | female pregnancy | 87 | 24 | 13.76 | 0.00035 | 0.071225 |
| GO:0034097 | response to cytokine | 626 | 145 | 98.99 | 0.00046 | 0.08368938 |
| GO:0051607 | defense response to virus | 180 | 46 | 28.46 | 0.00047 | 0.08368938 |
| GO:0034115 | negative regulation of heterotypic cell-... | 6 | 5 | 0.95 | 0.00051 | 0.08547 |
| GO:0045766 | positive regulation of angiogenesis | 67 | 23 | 10.59 | 0.00063 | 0.099715 |

Gene ontology term enrichment (Biological Process) of all genes significantly differentially expressed (FDR<0.05) in 5µm pores compared to control, but not 14µm pores compared to control or 5µm pores compared to 14µm pores.

**Table S4.** Gene ontology enrichment (Biological process) of Figure 2D (set 3)

| GO.ID | Term | Annotated | Significant | Expected | weight01Fisher | FDR |
| --- | --- | --- | --- | --- | --- | --- |
| GO:0050927 | positive regulation of positive chemotaxis | 8 | 3 | 0.13 | 0.00025 | 0.05225 |
| GO:0002268 | follicular dendritic cell differentiation | 2 | 2 | 0.03 | 0.00025 | 0.05225 |
| GO:0030036 | actin cytoskeleton organization | 353 | 17 | 5.59 | 0.00051 | 0.07106 |
| GO:0051481 | negative regulation of cytosolic calcium in concentration | 3 | 2 | 0.05 | 0.00074 | 0.07733 |
| GO:0002430 | complement receptor mediated signaling pathway | 4 | 2 | 0.06 | 0.00146 | 0.08718286 |
| GO:0002291 | T cell activation via T cell receptor contact with antigen bound to MHC molecule on antigen presenting cell | 4 | 2 | 0.06 | 0.00146 | 0.08718286 |
| GO:0061419 | positive regulation of transcription from RNA polymerase II promoter in response to hypoxia | 4 | 2 | 0.06 | 0.00146 | 0.08718286 |

Gene ontology term enrichment (Biological Process) of all genes significantly differentially expressed (FDR<0.05) in 5µm pores compared to 14µm pores, but not 14µm pores compared to control.

**Supplementary table 5.** Gene ontology enrichment (Molecular function) of Figure 2D (set 3)

| GO.ID | Term | Annotated | Significant | Expected | weight01Fisher | FDR |
| --- | --- | --- | --- | --- | --- | --- |
| GO:0015293 | symporter activity | 57 | 8 | 0.88 | 2.90E-06 | 0.0003161 |
| GO:0005096 | GTPase activator activity | 206 | 12 | 3.18 | 8.00E-05 | 0.00436 |
| GO:0015129 | lactate transmembrane transporter activity | 3 | 2 | 0.05 | 7.00E-04 | 0.02543333 |
| GO:0005078 | MAP-kinase scaffold activity | 6 | 2 | 0.09 | 0.0034 | 0.07412 |
| GO:0030506 | ankyrin binding | 6 | 2 | 0.09 | 0.0034 | 0.07412 |
| GO:0015171 | amino acid transmembrane transporter activity | 34 | 3 | 0.52 | 0.0047 | 0.08252857 |
| GO:0003779 | actin binding | 228 | 8 | 3.52 | 0.0053 | 0.08252857 |
| GO:0017137 | Rab GTPase binding | 110 | 6 | 1.7 | 0.007 | 0.08611 |
| GO:0008093 | cytoskeletal adaptor activity | 9 | 2 | 0.14 | 0.0079 | 0.08611 |
| GO:0008569 | ATP-dependent microtubule motor activity, minus-end-directed | 9 | 2 | 0.14 | 0.0079 | 0.08611 |

Gene ontology term enrichment (Molecular Function) of all genes significantly differentially expressed (FDR<0.05) in 5µm pores compared to 14µm pores, but not 14µm pores compared to control.

**Table S7.** RNA-seq QC.

| Condition | Replicate | Aligned | Assigned |
| --- | --- | --- | --- |
| ctrl | ctrl_1 | 46900000 | 25168155 |
| ctrl | ctrl_2 | 48500000 | 25271406 |
| ctrl | ctrl_3 | 44300000 | 23943174 |
| mig14 | mig14_1 | 53400000 | 28311460 |
| mig14 | mig14_2 | 45300000 | 25140336 |
| mig14 | mig14_3 | 46400000 | 26490266 |
| mig5 | mig5_1 | 45200000 | 24221809 |
| mig5 | mig5_2 | 49300000 | 26133154 |
| mig5 | mig5_3 | 50600000 | 26491917 |

Number of reads aligned and assigned to features in STAR and featureCounts per sample.

**Table S8.** HiCUP QC.

| Condition | Sample | Aligned | Valid_Pairs | Cis | Trans |
| --- | --- | --- | --- | --- | --- |
| ctrl | B1 | 285200000 | 82240869 | 20346475 | 34320173 |
| ctrl | C2 | 298900000 | 70896674 | 24487097 | 26631449 |
| ctrl | D3 | 249700000 | 90771248 | 31066409 | 45893185 |
| mig14 | Q4 | 313800000 | 79961656 | 30385557 | 24892331 |
| mig14 | R5 | 314300000 | 66324260 | 22255914 | 23613806 |
| mig14 | S6 | 313700000 | 65122979 | 22398913 | 20835777 |
| mig5 | X7 | 289300000 | 73974231 | 19287483 | 31260508 |
| mig5 | Y8 | 282900000 | 79694547 | 20971357 | 39606669 |
| mig5 | Z9 | 303500000 | 73563853 | 23595669 | 25564157 |

Number of aligned and valid reads, and cis trans ratio per sample.

**Table S9.** Hi-C replicate correlations.

| Condition | rep1 | rep2 | Correlation |
| --- | --- | --- | --- |
| ctrl | B1 | C2 | 0.99727658 |
| ctrl | B1 | D3 | 0.99552743 |
| ctrl | C2 | D3 | 0.99824302 |
| mig14 | Q4 | R5 | 0.99438484 |
| mig14 | Q4 | S6 | 0.99479017 |
| mig14 | R5 | S6 | 0.9990118 |
| mig5 | X7 | Y8 | 0.99839508 |
| mig5 | X7 | Z9 | 0.99806499 |
| mig5 | Y8 | Z9 | 0.99659289 |

Pearson correlation coefficients of the Hi-C library replicates. All biological replicates were highly correlated (R>0.994). The correlation coefficient was calculated across the whole genome with 1Mb bins.
